# Neocortical tau propagation is a mediator of clinical heterogeneity in Alzheimer’s disease

**DOI:** 10.1038/s41380-025-02998-y

**Published:** 2025-04-16

**Authors:** Anjalika Chongtham, Aarthi Ramakrishnan, Marissa Farinas, Diede W. M. Broekaart, Joon Ho Seo, Carolyn W. Zhu, Mary Sano, Li Shen, Ana C. Pereira

**Affiliations:** 1 Department of Neurology, Icahn School of Medicine at Mount Sinai, New York, NY USA; 2 Nash Family Department of Neuroscience, Friedman Brain Institute, Icahn School of Medicine at Mount Sinai, New York, NY USA; 3 Brookdale Department of Geriatrics and Palliative Medicine, Icahn School of Medicine at Mount Sinai, New York, NY USA; 4 James J. Peters VA Medical Center, Bronx, NY USA; 5 Department of Psychiatry, Alzheimer’s Disease Research Center, Icahn School of Medicine at Mount Sinai, New York, NY USA; 6 Sanford Grossman Interdisciplinary Program I Neural Circuitry and Immune Function, Icahn School of Medicine at Mount Sinai, New York, NY USA

**Keywords:** Neuroscience, Molecular biology

## Abstract

Heterogeneity in progression of clinical dementia obstructs the general therapeutic potential of current treatments for Alzheimer’s disease (AD). Though the mechanisms of this heterogeneity remain unclear, the characterization of bioactive tau species and factors that regulate their seeding behavior might give valuable insight as pathological tau is well correlated with cognitive impairment. Here, we conducted an innovative investigation into the molecular basis of widespread, connectivity-based tau propagation that begins in the inferior temporal gyrus (ITG) and spreads to neocortical areas such as the prefrontal cortex (PFC). Biochemical analysis of human postmortem ITG and PFC tissues revealed individual variability in tau seeding, which correlated with cognitive decline, particularly in the ITG, a region known for promoting accelerated tau propagation. Notably, this study presents the first evidence that site-specific phosphorylation and isoform composition of both aggregation-prone high-molecular-weight (HMW) tau and the relatively unexplored, yet potentially crucial in AD progression low-molecular-weight (LMW) tau significantly contribute to tau propagation and cognitive decline. Our findings underscore the importance of comprehensively considering diverse tau forms including both HMW and LMW tau species in understanding AD progression. Additionally, these results are the first to identify distinct morphological strains within the AD brain associated with differing seeding propensity, potentially enabling patient stratification based on their tau profile. Furthermore, RNA-seq analyses of gene expression patterns in the ITG revealed molecular heterogeneity associated with tau seeding potential. Patients with higher levels of seed-competent tau displayed greater impairments in synaptic and neural plasticity, and increased neuroinflammation. This multidisciplinary study offers novel insights into various molecular mechanisms driving AD progression, suggesting potential molecular targets for early intervention and improved patient subtyping, which is critical for developing precision medicine approaches.

## Introduction

Alzheimer’s disease (AD) is the most common neurodegenerative disorder characterized by progressive memory and cognitive functional decline [1]. Intraneuronal neurofibrillary tangles (NFTs) composed of abnormally hyperphosphorylated tau and extracellular amyloid-beta (Aβ) plaques are the defining neuropathological features of AD [2, 3]. In the neocortex, amyloid plaque build-up occurs over a decade or more before apparent cognitive symptoms, while neocortical tau accumulation timely coincides with the onset of cognitive symptoms and correlates with cognitive decline in patients [4, 5]. During AD progression, tau pathology appears to spread in a consistent spatiotemporal pattern throughout the brain along neuroanatomical connections, similar to those observed for the infectious prion protein [4, 6]. Furthermore, it has been recently shown that local Aβ-tau interactions in the inferior temporal gyrus catalyze widespread, connectivity-based tau propagation into neocortical regions, where degeneration can eventually give rise to dementia [7]. Despite these advances, uncertainties persist regarding whether the tau species involved in neuron-to-neuron transfer is fibrillar or not, and what its specific properties are. Achieving a more comprehensive understanding of the molecular basis of tau propagation is crucial for preventing the progression from early mild memory impairment to full cognitive deterioration and dementia.

Although the stereotypic spatiotemporal pattern of tau propagation may provide clues to the course of AD, accumulating evidence suggests that it is a heterogeneous disease caused by various pathophysiologic mechanisms and significant variability exists in the cognitive trajectories of affected individuals [8, 9]. Some patients progress very slowly over more than a decade, while others experience rapid cognitive decline, reaching severe dementia scores within very few years after symptom onset [10]. However, the precise molecular signatures that differentiate slow and fast progressors remain undetermined. Although aberrations in the regulation of gene expression patterns may contribute to disease progression [11], the exact causative factors for cognitive decline in AD, frequently associated with various co-pathologies [12], are still unclear. Bulk RNA-seq and network-based analysis demonstrate vulnerability of certain pathways to tau pathology, including microtubule-related pathways, synapse function, heat-shock response, and autophagy [13–15]. Despite this progress in the AD field, the heterogeneity of transcriptional responses associated with tau pathology has not been fully resolved. Currently, the field lacks a clear understanding of the precise molecular events and biological pathways that can lead to diverse rates of disease progression. Further, current AD drug trials do not account sufficiently for disease heterogeneity in trial design, impeding the development of effective drugs and personalized medicine approaches guided by tailored cognitive interventions [16]. A randomized clinical trial for anti-amyloid therapy, TRAILBRAZER-ALZ, in which patients were stratified by tau pathology, revealed a better drug response in the group with low-medium tau load. This study demonstrates the importance of taking tau pathology into account in clinical trials [17].

In this study, we aimed to decipher the molecular mechanisms driving heterogeneity in AD progression that remains a critical knowledge gap precluding rational therapeutic design. Based on previous findings and considering the close association between tau deposition and cognitive impairment in AD, we hypothesized that heterogeneity in cognitive trajectories may be explained by variation in the regional distribution of different tau species within the brain and their biochemical properties related to spread between communicating neurons [18, 19]. To investigate this, we analyzed postmortem brain samples from two key regions involved in tau spread, the inferior temporal gyrus (ITG) and prefrontal cortex (PFC). We characterized the biochemical and biophysical features of abnormal tau species, focusing on those that may influence its propagation and best predict AD progression. Additionally, we investigated transcriptomic profile alterations in the ITG associated with tau pathology and cognitive decline using RNA sequencing for molecular characterization of patients displaying tau and clinical heterogeneity. The present study integrated tau biochemistry with transcriptional alterations to elucidate the molecular mechanisms underlying cognitive decline in AD, which is critical for advancing future therapeutic interventions and development of personalized treatments.

## Materials and methods

### Human brain samples and lysate preparation

Frozen inferior temporal gyrus (ITG, Brodmann area 20) and prefrontal cortex (PFC, Brodmann area 9) brain tissues, consisting of grey matter from twenty subjects with AD were obtained from Banner Sun Health Research Institute (cohort 1, n = 10) and Mount Sinai Brain Bank (NIH NeuroBioBank) (cohort 2, n = 10). In addition, frozen brain tissues from the ITG and PFC of cognitively normal individuals were used as control. The demographic characteristics of the subjects are shown in Supplementary Table 1. Specific criteria used for selecting the AD cases were (1) Clinical diagnosis with AD; (2) Availability of MMSE (cohort 1) and CDR-SOB scores (cohort 2) for at least three visits at the clinic; (3) Braak neurofibrillary tangles (NFTs) stage V or VI with neocortical involvement; and (4) Minimal concurrent pathologies that can cause dementia. We calculated the linear estimate of the rate of cognitive decline or disease progression, extrapolated longitudinally from MMSE and CDR-SOB scores. The rate of cognitive decline was determined by calculating the slope of the linear regression line fitted to each subject’s MMSE or CDR-SOB score trajectory over time.

For biochemical studies, frozen human brain tissue (~300 mg) was homogenized in 5 volumes (w/v) of ice-cold PBS containing protease and phosphatase inhibitors (cOmplete mini and PhosStop, Roche Life Science, Burgess Hill, UK). The lysate was then centrifuged at 10,000 g for 10 min at 4 °C. The resulting supernatant was collected, and protein concentration was measured using the bicinchoninic acid (BCA) assay (Thermo Scientific Pierce, #23225). Additionally, total tau concentration in the samples was determined using an ELISA assay.

### Tau seeding assay

Tau seeding activity of human brain tissue lysate was determined using a previously published Tau RD P301S FRET Biosensor cell line (ATCC CRL-3275) stably expressing the repeat domain (RD) of tau containing the P301S mutation tagged to either CFP or YFP (TauRD-P301S-CFP/YFP) [20].

Biosensor cells were plated in 96-well plates at 5000 cells in 200 µL culture media per well and incubated at 37 °C. The following day, PBS-soluble brain lysate (8 ng of total tau quantified by total tau ELISA) was incubated with Lipofectamine 2000 (final concentration 1% vol/ vol) in opti-MEM (final volume of 50 μl per well) for 20 min at RT before being added to the cells. Transfection complexes remained in wells for 48 h. Cells were then washed once with PBS and fixed with 4% paraformaldehyde for 15 min. Confocal images were obtained using the 40× oil immersion lens with 405 (DAPI) and 488 (FRET) nm laser lines.

#### Image analysis

Percent FRET-positive cells were determined by running the captured images through the positive cell detection function in the QuPath 0.3.1 software with thresholds set at 8 units for detection of nuclei and 90 units for FRET positive cells. Total number of cells was determined based on the number of DAPI-stained nuclei and cell boundaries were determined using a cell expansion parameter of 5 µm from the nucleus. Measurements were averaged across six images per brain tissue sample. For the semi-quantitative assessment of aggregate morphology, we used QuPath 0.3.1 software to measure aggregate size and classified blinded images based on previously described prionoid tau aggregate morphologies [21, 22].

#### Patient classification

We classified AD patient samples as low, intermediate, and high seeders according to the percent-FRET-positive cells obtained from ITG tau seeding, which enabled percentile calculations. We found normal distribution in the study cohort, which allowed classification of the twenty AD cases into three groups (six low seeders, six intermediate seeders and eight high seeders). The % FRET-positive cells were <39.1 for low seeders and >57.3 for high seeders. To evaluate our ability to stratify subjects based on tau seeding activity, we assessed reproducibility using the Intraclass Correlation Coefficient (ICC) with a two-way random-effects model and absolute agreement. The average measurement of ICC was 0.987 (95% CI: 0.975 to 0.994), indicating that 98.7% of the total variability can be attributed to differences between subjects, with minimal variability within subjects.

### Total tau ELISA

Nunc MaxiSorp ELISA plates (BioLegend; 423501) were coated with Tau-5 (Invitrogen, #AHB0042; 0.5 µg/mL) in ELISA Coating Buffer (BioLegend; 421701) overnight at 4 °C. Plates were washed twice with PBST (0.05% Tween 20), blocked with 1% BSA/PBS for 2 h at RT, washed again and incubated with recombinant human tau standards (BioLegend; #842501) and brain samples diluted 1:100 in 1% BSA/PBS for 2 h at RT. After washing 4× with PBST, biotinylated BT2 + HT7 (Invitrogen, #MN1010B, #MN1000B; 0.25 µg/mL) was added for 45 min at RT followed by washing 6× with PBST. Streptavidin-HRP (BioLegend; 405210) was diluted 1:10,000 in 1% BSA/PBS and added to the plates for 30 min at RT. After washing 8× with PBST, plates were developed with TMB, stopped with 1N H_2_SO_4_, and read at 450 nm.

### pS396-tau ELISA

Phospho-tau concentrations in the brain samples were determined by an indirect ELISA using human pS396 tau peptide (Abcam; #ab226770) as standards. Briefly, Nunc MaxiSorp ELISA plates were coated with standard peptide and samples diluted 1:100 in PBS and incubated overnight at 4 °C. Plates were washed twice with PBST (0.05% Tween 20) and blocked with 1% BSA/PBS for 2 h at RT. After washing, pS396 antibody (Invitrogen; #44-752G) was biotinylated using a kit (Abcam; ab201795), added to the plates, and incubated overnight at 4 °C. The next day, plates were washed 4× and Streptavidin-HRP was added for 30 min at RT. Plates were then washed 6× with PBS, developed with TMB, stopped with 1N H_2_SO_4_, and read at 450 nm.

### SDS-PAGE and western blot

Brain lysates were adjusted to 10 μg total protein measured using a BCA assay (Thermo Scientific Pierce, #23225), heated for 5 min in sample buffer and resolved by SDS-PAGE on 4–20% polyacrylamide stain-free gels (Bio-Rad, #5678095) in Tris-glycine-SDS buffer. After transfer and blocking, membranes were probed with phospho-tau antibodies anti-pT181-tau (Thermo Fisher, #MN1050), anti-T217-tau (Thermo Fisher, #44-744), anti-pT231-tau (Thermo Fisher, #35-5200), anti-pS396-tau (Invitrogen; #44-752G); anti-pS396/pS404 or PHF1 (kind gift from P. Davies), Tau-13 (Biolegend, #835204), anti-3R-tau (Millipore, #05-803) and anti-4R-tau (Cosmo Bio, #CAC-TIP-4RT-P01) antibodies, antibodies against synaptic markers anti-PSD95 (Cell Signaling Technology, #36233) and anti-synaptophysin (Cell Signaling Technology, #4329). After overnight incubation at 4 °C, membranes were washed in PBS with 0.1% Tween 20 (PBST) and reacted with appropriate secondary antibodies for 1 h at RT. After washing again in PBST, membranes were scanned with the Bio-Rad ChemiDoc MP imaging system. Intensities of proteins bands were quantified by ImageJ analysis software (National Institutes of Health, USA) and normalized to total protein.

### Mediation analysis

We utilized the R package ‘mediation’ to assess whether the synaptic proteins PSD-95 and synaptophysin are mediators of tau seeding and cognitive decline. The equation for this relationship can be denoted as X ->M ->Y, where X represents tau seeding, M represents proteins associated with synaptic status, and Y represents the rate of cognitive decline (slope of the linearized MMSE or CDR-SOB score trajectories). The total effect of X on Y can be represented as Y~X. The direct effect of X on Y after taking into account the indirect effect of M can be represented as Y~X + M. The mediation effect is the difference between the total effect and the direct effect.

### RNA-Seq

Total RNA was isolated from eight control and twenty AD ITG brain tissue samples using PureLink RNA Mini Kit (Invitrogen, #12183018A). rRNA depletion using QIAseq® FastSelect™ rRNA HMR kit (Qiagen), and RNA sequencing libraries were constructed at Genewiz (South Plainfield, NJ, USA) with the NEBNext Ultra II RNA Library Preparation Kit for Illumina. Image analysis and base calling were conducted by the HiSeq Control Software (HCS). After extraction of gene counts using feature Counts from the Subread package v.1.5.2, downstream differential expression analysis was performed using DESeq2 package (v1.6.3). Genes with adjusted p-values < 0.05 and absolute log2FC > 1 were called Differentially Expressed Genes (DEGs) for each comparison.

#### GO enrichment analysis

Ingenuity Pathway Analysis (IPA; Qiagen) software was used to analyze the DEGs and identify canonical pathways. For the IPA analysis, we included the genes that had log2FC > 1 and Padj < 0.05.

#### Cell-type proportion analysis

The cell-type proportion of RNA-seq data used in this study was estimated using the brain cell-type marker signatures provided by the BRETIGEA R package [23]. The RNA–seq data was also normalized by brain cell type using the default parameters and calculated surrogate cell-type proportion (SPV) estimates.

#### SynGO annotation

For analysis of DEGs associated with synaptic localization and function, and to characterize synaptic transmission pathways, GO analysis was performed using the SynGO knowledgebase for the synapse [24]. The brain expressed background gene set was employed to identify the enriched synaptic components in DEGs at 1% FDR.

#### g:Profiler

To perform Enrichment analysis g:Profiler [25], a web server for gene ontology analysis, Ensembl version 108, was used. An ordered list of all protein coding DEGs was used as input from the comparisons (i) high seeders versus control and (ii) high seeders versus low seeders. From each gene list, we acquired the GO Biological Processes (BP) and Cellular Components (CC) that have a P-value of lower than 0.01 while using g:SCS multiple testing correction. A term size of 5 to 350 was applied to avoid inclusion of highly specific or highly general GO terms, respectively. Generic enrichment map file was loaded onto Cytoscape (version 3.9.1) with EnrichmentMap software (version 1.1.0) [26]. Enrichment map was generated by filtering genes by expression and using an FDR q-value cutoff of 0.1. Number of edges was determined using Jaccard+Overlap combined method with a cutoff value of 0.375. Afterwards, the AutoAnnotate app (version 1.3.5) in combination with WordCloud app was used to generate clusters.

#### Network analysis with WGCNA

The R WGCNA tutorial (https://github.com/edo98811/WGCNA_official_documentation) was referenced to construct a network using 20 Bulk RNA-seq samples from AD patients. Normalized and VST-transformed counts of the RNA-seq data were utilized. A co-expression similarity matrix was generated by computing the absolute values of the Pearson correlation coefficients between expression profiles of pairs of genes in the dataset. An adjacency matrix was derived from the co-expression similarity matrix by raising all values to a power of 3. This matrix provided the measure of network interconnectedness for pairs of genes. The soft-thresholding power of 3 was determined using pick-Soft Threshold function. Topological overlap matrix (TOM) was generated using the transformed adjacency matrix. Average linkage hierarchical clustering was applied on the TOM matrix to visualize the network. Modules were determined using the blockwiseModules function, where minModuleSize was set to 30 and mergeCutHeight was set to 0.5. The maxBlockSize was set to 40,000. Module eigenegenes were computed using the 1st principal component of the modules to summarize the profile of each module. Module eigengenes were correlated to slope values representing AD progression in patients. To identify causal regulators of WGCNA-derived modules, ARACNe-AP (https://github.com/califano-lab/ARACNe-AP) was run on VST expression values obtained for genes in each module identified by WGCNA [27]. This step allowed us to identify the most specific connections within each module. The human regulatory factors used in ARACNe-AP were extracted from https://humantfs.ccbr.utoronto.ca/download.php. Subsequently, we utilized the Cytoscape plugin cytoHubba [28] to rank nodes by degree within each ARACNe-AP network and identify key driver genes.

### Statistical analysis

All statistical tests were performed with GraphPad Prism 8 and SPSS Statistics 30.0.0.0 (IBM Corporation, Armonk, NY). Data are expressed as mean ± SEM, with statistical significance being determined as *P* values generated with a 95% confidence interval. All tests were two-sided with statistical significance set a priori at *P* < 0.05. Spearman rank non-parametric correlation was performed to correlate different variables from individual patients. The Spearman correlation coefficient *r* and *P* values are shown in the figures. We used unpaired *t* test for comparison of variables between low and high seeders, accounting for both equal and unequal variances. For cell-type proportion analysis, differences in gene expression were determined using two-way ANOVA with Sidak’s multiple comparisons test.

## Results

### Tau seeding in AD ITG increases with faster cognitive decline

**Fig. 1:**
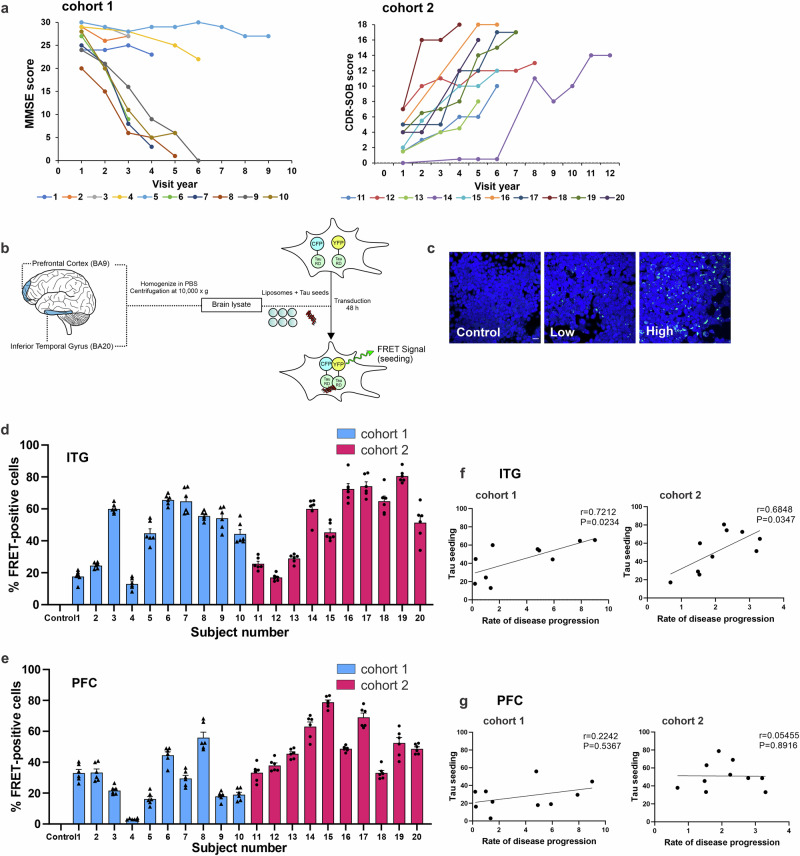
Tau seeding bioactivity correlates with cognitive decline in AD. **a** Graphs showing dementia progression in patients from Banner Sun Health Research Institute (cohort 1) and Mount Sinai ADRC (cohort 2). A line corresponds to a single patient and each dot to a visit at the research clinic. The patients were evaluated for their MMSE (cohort 1) or CDR-SOB (cohort 2) scores at each visit. **b** Schematic representation of the in vitro FRET-based tau seeding assay. **c** Representative 40× confocal images of FRET-biosensor cells showing no seeding, low and high tau seeding after treatment with a control and AD soluble brain lysates, with FRET-positive tau inclusions shown in bright green and nuclear DAPI staining in blue. Scale bar, 20 µm. **d**, **e** Graphs representing tau seeding activity (% FRET-positive cells) induced by ITG **d** and PFC **e**. Brain lysates from one cognitively unimpaired control and twenty patients were used. **f**, **g** Tau seeding induced specifically by ITG lysates **f** were significantly correlated with the rate of cognitive decline in both cohorts while the correlation between seeding by PFC lysates and cognitive decline was not significant **g**, determined using a two-tailed Spearman’s rank non-parametric test, *P* and *r* values are indicated on each plot.

To study tau biochemical diversity in the human AD brain, we characterized two cohorts of patients with AD (n = 20 total) based on clinical and pathological features (Supplementary Table 1). Two brain regions important for tau propagation and cognition were analyzed, the ITG (Brodmann area 20), featuring a connectivity profile well suited to accelerate tau propagation [7], and the PFC (Brodmann area 9), involved in executive function and affected in later stages of AD [29]. All participants were diagnosed antemortem and postmortem with AD and had neuropathological patterns of advanced AD (Braak V/VI). We measured the linear estimate of the rate of clinical progression, extrapolated longitudinally from the Mini-Mental State Examination (MMSE) and Clinical Dementia Rating Scale Sum of Boxes (CDR-SOB) scores over a minimum of three annual visits at Banner Sun Health Research Institute and Mount Sinai Alzheimer’s Disease Research Center (ADRC), respectively. The study participants had different rates of cognitive decline, indicative of the clinical heterogeneity of AD progression (Fig. 1a).

Templated proteopathic tau seeding is an early and robust marker of tauopathy, preceding any detectable histopathological changes [20], and occurs in a time- and concentration- dependent manner [30]. Total brain lysates prepared from frozen postmortem ITG and PFC tissues were tested for seeding activity using an ultrasensitive and specific fluorescence resonance energy transfer (FRET)-based biosensor assay [20]. The cell-based assay measures the ability of tau seeds to induce misfolding and aggregation of naïve monomeric tau. This is based on stable expression of repeat domain of tau (TauRD) with the disease-associated P301S mutation fused to either CFP (TauRD-CFP) or YFP (TauRD-YFP) in human HEK 293 cells. A FRET signal can be generated when exogenous tau seeds delivered to the cell interior with phospholipids (lipofectamine) trigger the aggregation of the endogenously expressed TauRD-CFP/YFP (Fig. 1b, c). When the tau biosensors were treated with control brain lysate, no FRET-positive inclusions were seen at 48 h after the addition of lysate. However, upon treatment with AD brain lysates containing the same amount of tau measured by total tau ELISA, intra-cellular TauRD-CFP/YFP aggregates were observed (Fig. 1c). We then quantified tau seeding based on the % FRET-positive cells. Even within a similar Braak stage (V/VI) and despite adding equal tau amounts to the seeding assay, not all the AD cases followed the same tau seeding pattern. We found considerable variability in tau seeding capacity of both brain regions across patients, highlighting the complex heterogeneity of AD (Fig. 1d, e). We next tested for correlation of tau seeding bioactivity with the rate of disease progression. Seeding activity and the rate of cognitive decline significantly positively correlated, particularly in the ITG but not in the PFC (Fig. 1f, g). Furthermore, our data show the age of disease onset, a potential marker of disease severity, and age at death correlated specifically with ITG-induced tau seeding (Supplementary Fig. 1a, b).

### Tau seeding propensity determines aggregate morphology and increases with phospho-tau levels

**Fig. 2:**
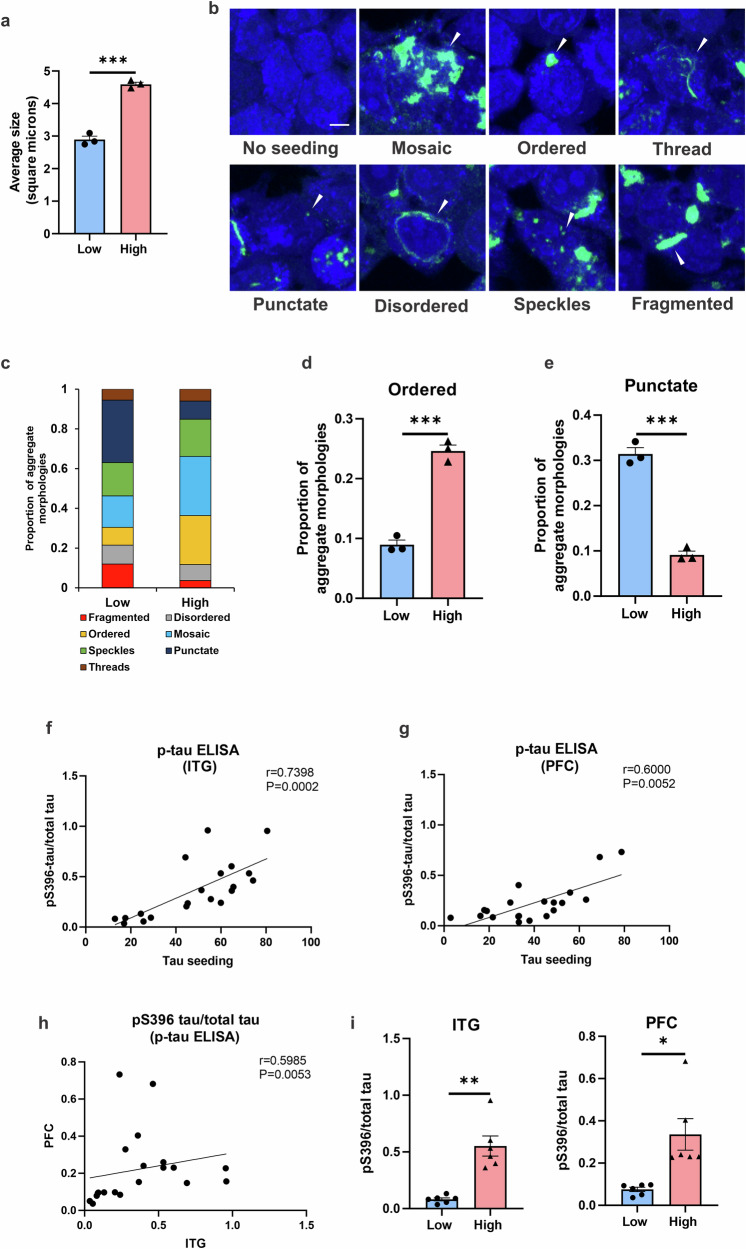
AD brain lysates with elevated phospho-tau induce more tau seeding and higher order, more complex inclusion morphology. **a** High seeders generated larger aggregates compared to low seeders in tau FRET-biosensor cells. Data are presented as mean ± SEM; n = 3 AD cases per group. ***P < 0.001 (t test). **b** Several aggregate phenotypes, mosaic, ordered, thread, punctate, disordered, speckles and fragmented (arrowheads) were delineated for the semi-quantitative assessment of aggregate morphology. Scale bar, 5 µm. **c** The relative proportion of each morphological phenotype (high versus low seeders) defined in **b** was quantified. **d**, **e** High seeders generated more ordered **d** and less punctate inclusions **e** compared to low seeders. Data are presented as mean ± SEM; n = 3 AD cases per group. ***P < 0.001 (t test). **f**, **g** Phospho-tau (pS396) levels in both ITG **f** and PFC **g** brain lysates show a significant correlation with tau seeding. **h** Intensities of pS396 tau in the ITG and PFC were correlated, showing a significant association. Correlations were performed using a two-tailed Spearman’s rank non-parametric test, *r* and *P* values are indicated on the plots. n = 20 individual human participants. **i** Intensities of pS396 tau were increased in both the ITG and PFC brain tissues of high seeders compared to low seeders. Data are presented as mean ± SEM; n = 6 AD cases per group. *P < 0.05, **P < 0.01 (t test).

To understand why tau seeding assessed by the FRET biosensor assay varies across individuals, we sought to determine the size and morphology of ITG-induced cytoplasmic inclusions. To this end, inclusion morphology was semiquantitatively analyzed according to a cell-based strain isolation system [21] that allows the morphological classification of tau inclusions within biosensor cells treated with tau seeds derived from different human tauopathy brain homogenates to delineate biochemically distinct prion-like strains of tau aggregate conformers. Using percentile distribution, we first classified the patient population into subgroups according to ITG seeding propensity (low seeders with % FRET- positive cells <39.1 and high seeders with % FRET-positive cells >57.3), followed by characterization of inclusion size and morphology. Our results revealed that aggregate size was significantly increased in high seeders compared to low seeders (Fig. 2a). Additionally, we identified multiple morphologic ‘strains’ of aggregates that formed in the tau RD reporter cell line in response to tau seeds. Our results suggest that tau seeds in low seeders are limited in their ability to induce aggregation and may be less able to self-assemble into higher-order, more complex structures and instead form more punctate phenotype (Fig. 2b–e). To further address the variation in tau seeding potential, we characterized tau seeds present in the brain lysates using biochemical assays. We measured the levels of phosphorylated tau at epitope S396 (pS396 tau) by human tau-specific enzyme-linked immunosorbent assay (ELISA) in the brain lysates and correlated these measures with their seeding capacity measured by the FRET-based-biosensor assay. Our findings indicate that tau seeding significantly positively correlated with the amount of p-tau in both brain regions (Fig. 2f, g), supporting the idea that tau seeds are predominantly composed of hyperphosphorylated tau species [20, 31]. Additionally, we found significant positive correlation between the amount of pS396 tau in the ITG and PFC (Fig. 2h). Compared to low seeders, high seeders had elevated levels of pS396 tau in both their ITG and PFC brain regions (Fig. 2i). We speculate that elevated tau phosphorylation in the ITG propagation hubs may lead to increased seeding activity and transcellular spread of pathological tau seeds into functionally connected cortical areas.

### Specific AD-relevant phospho-tau epitopes are associated with tau seeding

**Fig. 3:**
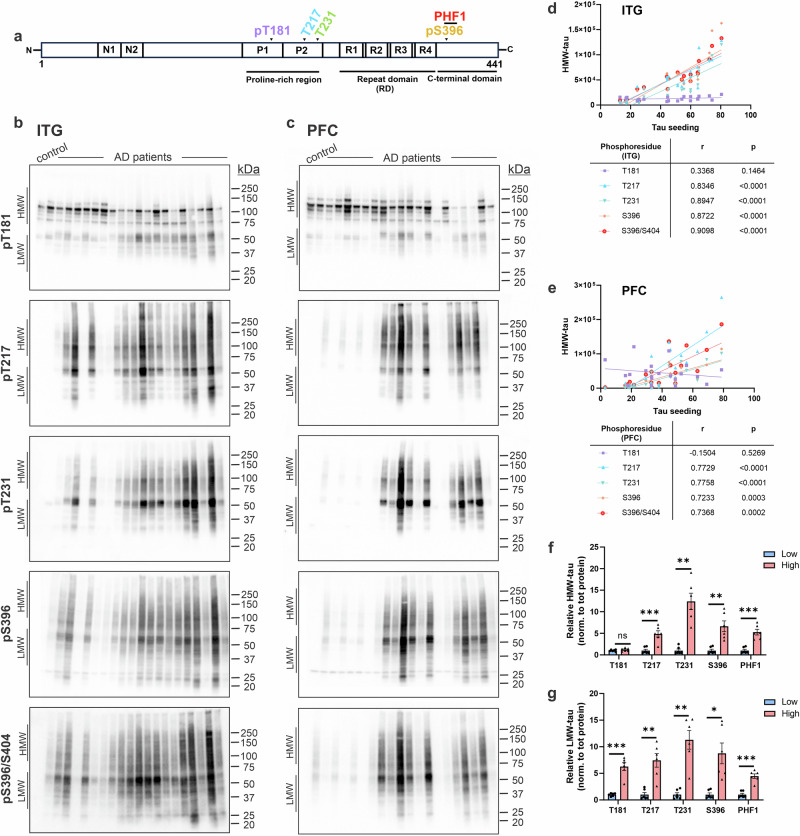
Phosphorylation of HMW-tau at specific sites associated with AD correlates with tau seeding. **a** Schematic diagram of the epitopes of tau antibodies used for western blot analysis. **b**, **c** Representative western blots of ITG **b** and PFC **c** brain lysates from twenty AD cases (subject no. 1–5, 11–15, 6–10, 16–20) probed with the indicated site-specific and phosphorylation dependent anti-tau antibodies. **d**, **e** Immunoblots were analyzed by densitometry, normalized by total protein and the levels of phosphorylated HMW-tau in the ITG **d** and PFC **e** brain tissues were correlated with seeding. Two-tailed Spearman’s rank non-parametric correlation tests were used, and *r* coefficient and *P* values are indicated on the tables. **f**, **g** Quantification of HMW and LMW-phospho tau levels in the ITG of low (blue bars) and high seeders (pink bars), represented as fold change of low seeders. Data are presented as mean ± SEM; n = 6 AD cases per group. *P < 0.05, **P < 0.01, ***P < 0.001 (t test).

We next sought to establish the correlation between seeding and the amount of site-specific, hyperphosphorylated oligomeric tau in the ITG and PFC brain tissues. In humans, phosphorylation of specific serine and threonine residues of tau correlates spatiotemporally with AD progression and can be employed to stage the cytopathological status of the disease [3]. In order to increase understanding of the band profile of p-tau and identify the most biologically relevant conformers associated with seeding and disease progression, brain lysates were blotted and analyzed using several phospho-site-specific anti-tau antibodies directed against the proline-rich (pT181 and T217), core (pT231) and C-terminal domains (pS396 and pS396/pS404 [PHF1]), which are hyperphosphorylated in AD (Fig. 3a–c). We found that the band patterns of tau in the brain samples are dependent on the site-specific antibodies used for the analysis. Our data indicate that phosphorylation of high-molecular-weight (HMW) tau species (SDS and reducing agent resistant tau forms; 65 kDa upwards) appears to be heterogeneous across individuals, with high seeders enriched in HMW-tau. The amount of HMW-tau phosphorylation in both the ITG and PFC brain samples at all epitopes except T181, significantly positively correlated with seeding (Fig. 3d, e). We observed an intense 130 kDa pT181 band in both control and AD samples, suggesting the possibility of a nonspecific reaction or the presence of pT181 and other proteins with similar molecular weight. Additionally, there were significant positive correlations between the levels of HMW-tau phosphorylation at all epitopes tested (except T181) in ITG versus PFC (Supplementary Fig. 2), suggesting that the extent of site-specific phosphorylation of HMW-tau in the ITG may impact seeding and accelerated propagation along neuroanatomical connections.

Compared with low seeders, phosphorylation level of ITG HMW-tau was increased at all analyzed phosphorylation sites, except T181, in high seeders, with 4.9, 12.4, 6.6 and 5.3-fold increase for pT217, pT231, pS396 and S396/S404, respectively (Fig. 3f). Furthermore, we saw lower bands corresponding to low-molecular-weight (LMW) tau species (monomers and proteolysis products), ranging from 65 to 20 kDa with antibodies specifically directed to the carboxy-terminal epitopes (pS396 and PHF1), thus suggesting the presence of truncated or cleaved forms of tau containing the C-terminal region. Our immunoblotting data indicate increased phosphorylation of ITG LMW-tau species in high seeders compared to low seeders at all studied phosphoepitopes, with 6.3, 7.4, 11.3, 8.8 and 4.5-fold increase for pT181, pT217, pT231, pS396 and pS396/S404, respectively (Fig. 3g). Taken together, these results support the notion that hyperphosphorylation of both HMW and LMW-tau species at specific sites in the ITG brain region may play an important role in tau pathogenesis and AD progression.

### Tau isoforms are differentially expressed in AD brains and their expression levels correlate with seeding

**Fig. 4:**
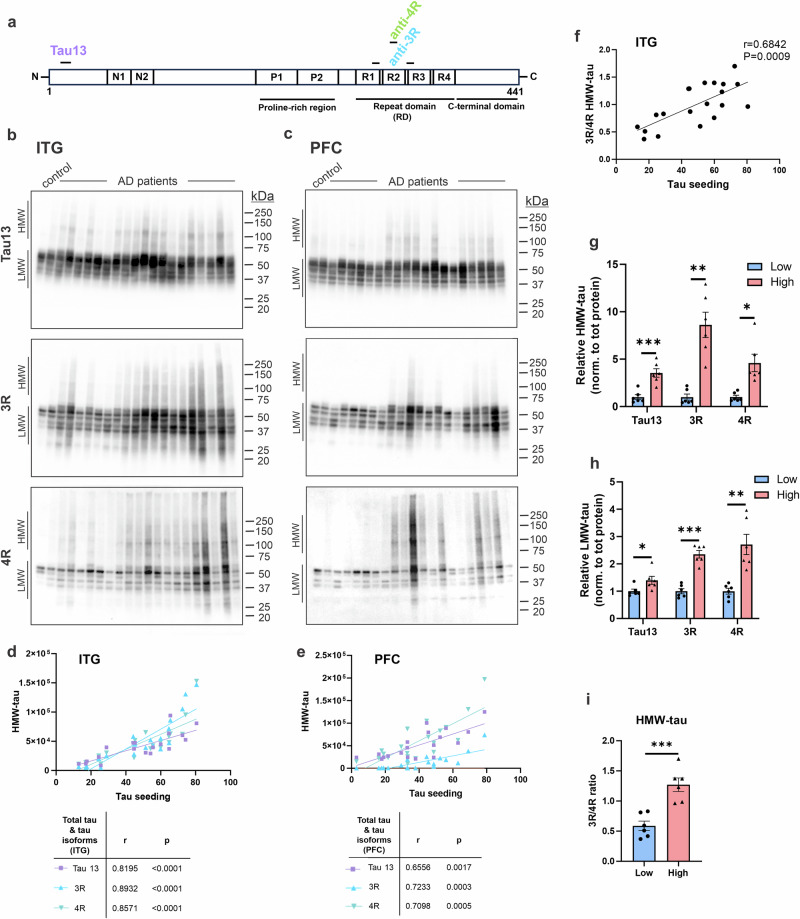
Tau seeding correlates with oligomeric 3R and 4R tau levels. **a** Schematic diagram of the epitopes of tau antibodies used for western blot analysis. **b**, **c** Representative western blots of ITG **b** and PFC **c** lysates from twenty AD cases (subject no. 1–5, 11–15, 6–10, 16–20) developed with total tau (Tau13), anti-3R and 4 R tau antibodies. **d**, **e** Immunoblots were densitometrically quantified, normalized by total protein and HMW-3R and 4 R tau levels in the ITG **d** and PFC **e** were correlated with seeding. Two-tailed Spearman’s rank non-parametric correlation tests were used, and *r* coefficient and *P* values are indicated on the tables. **f** Correlation of the ratio of 3R-tau/4R-tau in the ITG from twenty AD cases with seeding is shown. A two-tailed Spearman’s rank non-parametric correlation test was used, and *r* coefficient and *P* values are indicated on the plot. **g**, **h** Quantification of HMW and LMW-total tau and isoform levels in the ITG of low and high seeders, represented as fold change of low seeders. Data are presented as mean ± SEM; n = 6 AD cases per group. *P < 0.05, **P < 0.01, ***P < 0.001 (t test). (**i**) Relative 3 R/4 R HMW-tau ratio in the ITG from six low and six high seeders was detected by immunoblots. Results represent the mean ± SEM; ***P < 0.001 (t test).

In the adult human brain, tau is expressed as six different isoforms that vary in the number of N-terminal inserts (0N, 1N, or 2N) and C-terminal repeat domains (3R or 4R) [32]. Understanding how different tau isoforms contribute to tau seeding and disease progression is imperative, because distinct isoforms and isoform composition can influence the process of tau aggregation and propagation in AD [33, 34]. To determine the expression profiles of tau and its isoforms in the brain lysates and investigate the potential role of the C-terminal repeat domain (RD) in seeding and disease progression, we measured the levels of total tau, 3R-tau, and 4R-tau in the AD brain lysates by western blots and analyzed their correlation with seeding (Fig. 4a–c). We found strong positive correlations between seeding and HMW total tau (Tau13), 3R and 4R-tau levels in both ITG and PFC brain lysates (Fig. 4d, e). A significant positive correlation was also observed between the ratio of 3R/4R tau and seeding, suggesting that disease progression may contribute to the change in the ratio of 3R and 4R-tau in the ITG or vice versa (Fig. 4f). We compared the total tau, 3R and 4R-tau in the ITG lysates with different seeding potential and found that HMW-tau levels were significantly elevated in high seeders compared to low seeders, with 3.5, 8.6 and 4.6-fold increase total tau (Tau13), 3R and 4R, respectively (Fig. 4g). We also observed a substantial increase in the LMW-3R and 4R tau expression levels by 2.4 and 2.7-fold respectively, and a slight increase in LMW total tau expression by 1.4-fold in the high seeders (Fig. 4h). Furthermore, the high seeders displayed an increased 3R/4R HMW-tau ratio compared to low seeders (Fig. 4i).

### Specific tau phosphorylation sites and repeat domains are associated with AD progression

**Fig. 5:**
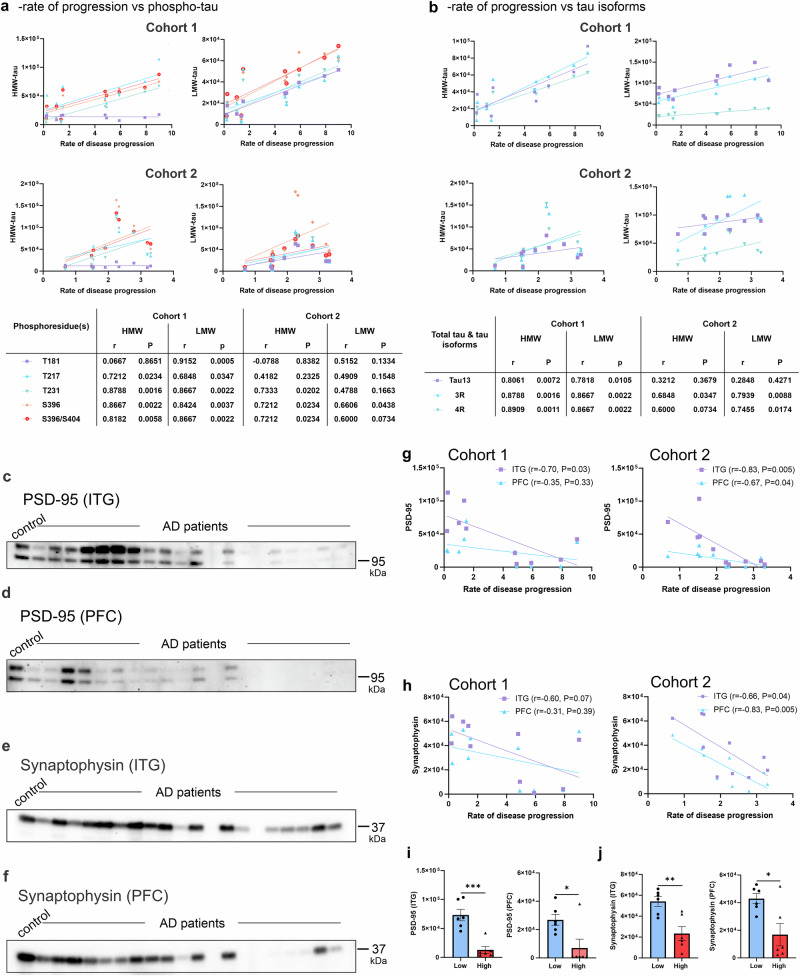
HMW and LMW-phosphorylated tau and isoforms are associated with cognitive decline. **a**, **b** The levels of phosphorylated HMW and LMW-tau **a**, and HMW and LMW total tau, 3 R and 4 R isoforms **b** in the ITG of cohorts 1 and 2 (twenty AD cases) determined by western blots were correlated with the rate of cognitive decline. Two-tailed Spearman’s rank non-parametric correlation tests were used; *r* and *P* values are indicated on the tables. **c**-**f** Representative western blots of ITG and PFC lysates from a control and twenty AD cases (subject no. 1–5, 11–15, 6–10, 16–20) developed with antibodies against PSD-95 **c**, **d** and synaptophysin **e**, **f**. **g**, **h** Immunoblots were densitometrically quantified, normalized by total protein and the levels of synaptic proteins in the ITG and PFC lysates were correlated with the rate of cognitive decline. Correlations were performed using a two-tailed Spearman’s rank non-parametric test, and *r* and *P* values are indicated on the plots. **i**, **j** Quantification of PSD-95 and synaptophysin levels in the ITG and PFC of low and high seeders. Data are presented as mean ± SEM; n = 6 AD cases per group. *P < 0.05, **P < 0.01, ***P < 0.001 (t test).

Soluble phosphorylated HMW-tau species can be robustly taken up, axonally transported, secreted and retaken up synaptically through connected neurons [35]. Therefore, we evaluated the potential role of HMW p-tau (pT181, pT217, pT231, pS396, pS396/S404), 3R and 4R tau isoforms in disease progression. We observed significant positive correlations between the phosphorylation levels of HMW-tau (except HMW pT181) in the ITG determined by immunoblots and the rate of cognitive decline (Fig. 5a). Additionally, among total tau and tau isoforms, our data show a significant positive correlation, specifically between HMW 3R-tau and the rate of cognitive decline in both cohorts (Fig. 5b). Conversely, the correlations between HMW p-tau and isoforms in the PFC and cognitive decline were either weak or not significant (Supplementary Fig. 3a, b).

Further, we found significant positive correlations between cognitive decline and LMW-tau levels for all phospho-sites tested, total tau, and isoforms in the ITG region of cohort 1, while in both cohorts, pS396-LMW tau and tau isoforms significantly correlated with cognitive decline (Fig. 5a, b). These findings validate that phosphorylation at specific sites and especially in the ITG brain region likely involves conformational changes impacting the seeding activity of tau that can influence its propagation and disease progression.

The aberrant accumulation and spread of pathological tau through synaptically connected neurons may cause synaptic damage and cognitive decline [36, 37]. We next explored whether synaptic protein changes in the ITG and PFC were associated with tau propagation and cognitive decline. Synapse loss and its associations with AD progression was determined via immunoblotting of two synaptic proteins, namely synaptophysin (presynaptic marker), a major integral membrane glycoprotein localized to presynaptic vesicles, and PSD-95 (postsynaptic density protein), a prominent postsynaptic scaffolding protein that, on the grounds of their differential role in the synaptic machinery, represent important candidates for investigation [38, 39]. We observed that the rate of cognitive decline was associated with both PSD-95 and synaptophysin protein levels in the ITG and PFC brain lysates (Fig. 5c–h). Inverse statistically significant correlations to cognitive decline were present for PSD-95 in the ITG of both cohorts 1 and 2 and in the PFC of cohort 2, i.e., reduced PSD-95 levels correlated with faster cognitive decline. Similarly, a decrease in synaptophysin concentration was associated with worsening cognition reflected by significant negative correlations between cognitive decline and the presynaptic marker in the ITG and PFC of cohort 2.

Correlative analysis between synaptic pathology and p-tau sites revealed that both PSD-95 and synaptophysin demonstrated significant negative correlation with the amount of HMW-tau phosphorylated at T231, pS396 and pS396/S404 (Supplementary Fig. 3c). Notably, PSD-95 significantly negatively correlated with HMW total tau and both 3R and 4R tau isoforms (Supplementary Fig. 3d, top). While synaptophysin showed some degree of a weak negative correlation with HMW total and 4R-tau, significant negative correlation was found between synaptophysin and 3R tau isoform (Supplementary Fig. 3d, bottom). Furthermore, compared to low seeders, high seeders had reduced synaptic markers in both ITG and PFC brain tissues (Fig. 5i, j), implicating tau seeding as a potential mediator of AD-related synaptic deficits.

Finally, we conducted a mediation analysis to assess whether PSD-95 and synaptophysin levels in the ITG mediate the relationship between tau seeding and cognitive decline. Our results revealed a significant mediation effect of PSD-95 (0.3169, p = 0.04) and synaptophysin (0.194, p = 0.012) on this relationship. In addition, a significant (p = 0.00089) negative (−0.68) correlation was observed between tau seeding and PSD-95 levels. These findings suggest that tau seeding may contribute via changes in markers of synaptic integrity, dysfunction and loss to cognitive decline.

### Tau pathology associated global gene expression changes in AD ITG

**Fig. 6:**
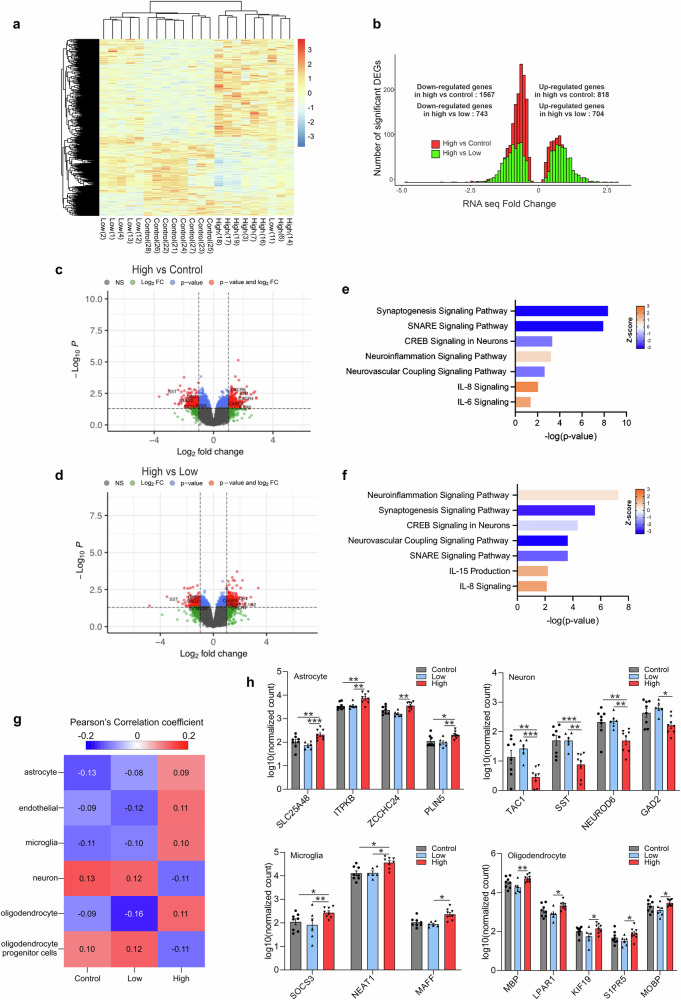
Transcriptional changes in AD ITG associated with tau pathology and disease progression. **a** Heatmap shows the clustering of fourteen AD (six low seeders and eight high seeders) and eight control ITG samples. Subject number is included in the parenthesis. Normalized expression levels of significant (P < 0.05; abs(log2FC) > 1.2) DEGs in low seeders vs control and high seeders vs control are shown. Z-score transformation is applied for each row. The color scale illustrates the expression levels: a deep red indicates higher expression while a dark blue signifies lower expression. **b** Histogram representing the number of DEGs (high seeders vs control in red, high vs low seeders in green, Padj < 0.05). **c**, **d** Volcano plots illustrating the distribution of DEGs between high seeders and control **c**, and high and low seeders **d**. The filtering criteria used were Padj<0.05, log2 (FC) > 1. **e**, **f** Canonical pathways derived from IPA gene ontology algorithms for significantly dysregulated genes in high seeders compared to control **e** and low seeders **f**. **g** Pearson correlation between marker gene expressions in six cell types in eight control, six low and eight high seeders, computed by averaging surrogate cell-type proportion values (SPVs) for the brain tissue samples after cell-type deconvolution by BRETIGEA. **h** Graphs showing the log10(normalized gene expression) of selected astrocyte, neuronal, microglial and oligodendrocyte marker genes in control, low and high seeders. Data represent the mean ± SEM; *P < 0.05, **P < 0.01, ***P < 0.001 (Two-way ANOVA, Sidak’s multiple comparisons test).

To examine the potential molecular basis for the observed differences in biochemical properties of tau across the AD brains, we employed whole transcriptome RNA-seq analysis to characterize the molecular signatures and transcriptional landscape in control and AD ITG brain tissues with differential tau seeding potential. Hierarchical clustering analysis was conducted to assess the expression profiles of differentially expressed RNA transcripts between cognitively unimpaired control and AD groups (low and high seeders) (Fig. 6a). Analyses of the transcriptomic data with a cut off of adjusted P < 0.05 identified 2385 (1567 down and 818 upregulated genes) and 1447 (743 down and 704 upregulated genes) DEGs in high seeders relative to control and low seeders, respectively (Fig. 6b). We used volcano plots to illustrate the distribution of DEGs and the significant DEGs were further subjected to core analysis for canonical pathway using Ingenuity Pathway Analysis (IPA; Qiagen) (Fig. 6c–f). The results demonstrated that several immune/inflammation-related genes such as CXCr4, IL1R2, CR1, CXCr6, and CASP4, and pathways, including neuroinflammation and interleukin (IL-6, 8, 15) signaling were predicted to be activated in high seeders. On the other hand, synapse-related genes such as SV2A, SYT1, GAD1, SST, and GAD2 that regulate synaptic vesicle secretion and glutamatergic neurotransmission, and functional categories such as synaptogenesis and SNARE signaling pathways were predicted to be inhibited in high seeders.

To understand better the brain cell-type specificity of transcriptomic changes in control and each AD subset (low or high seeders), we performed cell-type proportion analysis on the ITG brain samples using the brain cell-type marker signatures determined previously by cell-type specific sequencing [23] (Fig. 6g, h). Our analyses revealed similar cell type-specific gene expression profiles in control and low seeders with relatively high contribution of neurons and oligodendrocyte precursor cells (OPCs) accompanied by a low contribution of astrocytes, endothelial cells, microglia, and oligodendrocytes. In contrast, high seeders exhibited relatively low presence of neurons as well as OPCs combined with high presence of astrocytes, endothelial cells, microglia, and oligodendrocytes (Fig. 6g). Next, we analyzed the cell-type specificity of the key regulator genes of control and AD groups. As shown in Fig. 6h, high seeders had downregulated neuronal regulators (TAC1, SST, NEUROD6, GAD2) and upregulated genes in astrocytes (SLC25A48, ITPKB, ZCCHC24, PLIN5), microglia (SOCS3, NEAT1, MAFF) and oligodendrocytes (MBP, LPAR1, KIF19, S1PR5, MOBP). These unique patterns in high seeders are consistent with axonopathy, neuronal death and reactive gliosis indicative of immune system activation commonly seen in AD cases [40].

### Dysregulation of synaptic transmission pathways is closely associated with tau pathology

**Fig. 7:**
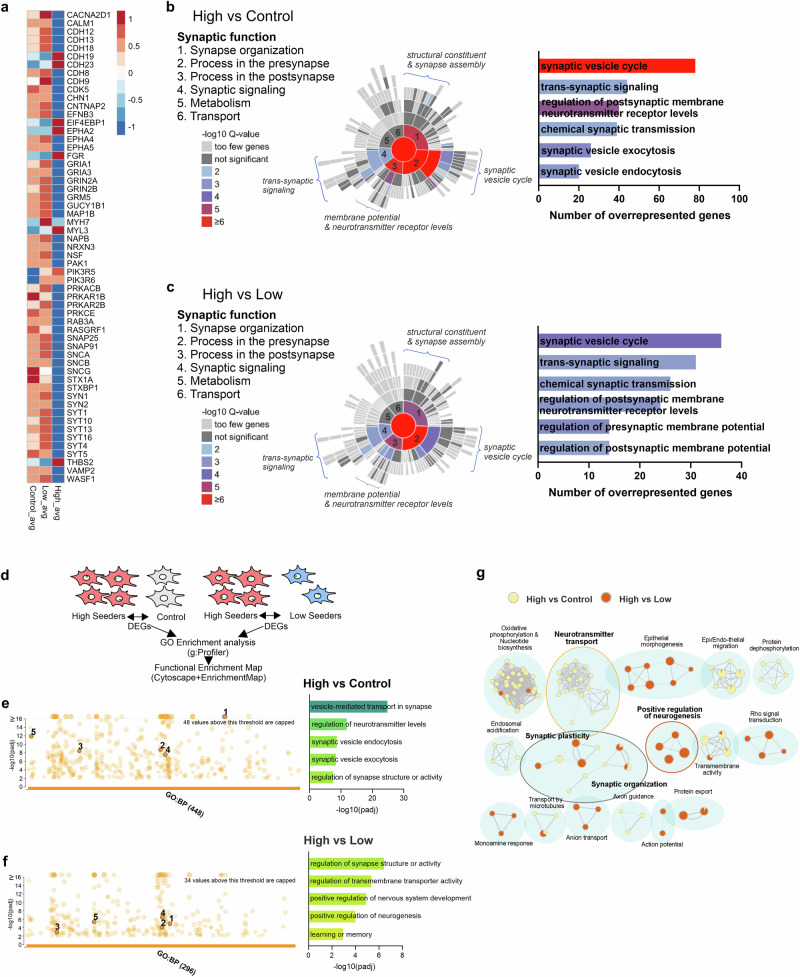
Transcriptional dysregulation of synaptic transmission pathways in AD ITG. **a** Heatmap depicting the expression levels of 57 synaptic genes in postmortem ITG averaged across replicates for control (n = 8), low (n = 6) and high (n = 8) seeders. **b**, **c** Sunburst plots representing enriched biological process terms in high versus control **b** and high versus low seeders **c**; colors represent enrichment values at 1% FDR. The bar plots highlight top six enriched terms for each comparison. **d** Overview of strategy to identify shared and unique pathways overrepresented in eight high seeders compared to eight control and six low seeders. **e**, **f** Manhattan plots of GO biological processes enriched in high seeders versus control **e** and high versus low seeders **f** for each cluster obtained using g:profiler. Colored circles denote significant terms (thresholds: g:SCS significance <0.01; GO term size 5-350). Top 5 nonredundant terms are highlighted. **g** Functional enrichment map. Nodes represent gene sets of GO biological processes; each node is color coded by cluster to illustrate shared and unique contributions.

With synaptic terms such as synaptogenesis and SNARE signaling being some of the top significantly downregulated pathways in the ITG of AD cases with increased tau seeding capability, we further examined the expression levels of synaptic genes among the different AD groups (low and high seeders) and cognitively unimpaired controls. As shown in Fig. 7a, many of the SNARE complex genes involved in presynaptic vesicle exocytosis, including SNAP-25, STX1A, SYT1 and VAMP2 were expressed at relatively low levels in high seeders compared to control and low seeders. Some genes encoding postsynaptic glutamate receptors, GABA receptors or anchoring proteins including GRIN2A, GRIN2B, GRIA1, GRIA3 and GRM5 were also relatively low in high seeders compared to control and low seeders. Using SynGO, a synaptic gene ontology database [24], we found significant enrichment of sixteen biological process terms (Fig. 7b, Supplementary Table 2) and eighteen cellular component (Supplementary Fig. 4a, Supplementary Table 2) terms in high seeders versus control. In addition, compared to low seeders, high seeders had eleven enriched biological process terms (Fig. 7c, Supplementary Table 3) and eighteen cellular component terms (Supplementary Fig. 4b, Supplementary Table 3). For both comparisons, top-level enriched biological process terms included synapse organization, process in the presynapse, process in the postsynapse and synaptic signaling, whereas metabolism and transport were underrepresented. We also found the highest enrichment scores corresponded with the synaptic vesicle cycle.

To visualize the shared and unique dysregulated pathways between high seeders versus control and high versus low seeders, we performed a functional enrichment analysis (Fig. 7d). Using g:Profiler [25], we generated a ranked list of significant Biological Processes for each comparison (Fig. 7e, f). We integrated these Gene Ontology (GO) terms using the EnrichmentMap tool via Cytoscape [26], in which nodes represent single GO terms and lines represent overlap between GO terms (Fig. 7g). Overall, the resulting EnrichmentMap indicates hubs of related nodes that are associated with neuronal functions, intercellular reorganization and signal transduction. Notably, neurotransmitter transport was enriched uniquely in the high versus control comparison (yellow), including the GO biological processes of vesicle-mediated transport in synapse (GO:0099003), regulation of neurotransmitter levels (GO:0001505) and synaptic vesicle endo- and exocytosis (GO:0048488, GO:0016079). GO terms related to the positive regulation of neurogenesis were found to be enriched solely in high versus low seeders (orange), including positive regulation of cell projection organization (GO:0031346), nervous system development (GO:0051962), neurogenesis (GO:0050769) and cell development (GO:0010720). Interestingly, biological processes related to synaptic plasticity and synaptic organization were enriched in both high versus control and high versus low seeders (Supplementary Table 4), reflecting similar pathways identified with IPA such as the synaptogenesis and SNARE signaling pathways. Multiple genes indicated by these pathways overlapped with the SynGO analysis, including CDK5, EFNB3, EPHA4, GRIN2A, GRIN2B, MAP1B and SNCA. Involvement of synaptic pathways was strengthened by analyzing cellular component terms (Supplementary Fig. 4c–e). Both comparisons were enriched for GO terms belonging to the presynaptic membrane, postsynaptic density and vesicle-related terms (Supplementary Fig. 4e). Taken together, these findings suggest that increase in tau seeding activity and disease progression is associated with disruption of transcriptional homeostasis of synapses and impairment of neurotransmission in the ITG brain region.

### Construction of gene co-expression networks and identification of distinct modules associated with cognitive decline

**Fig. 8:**
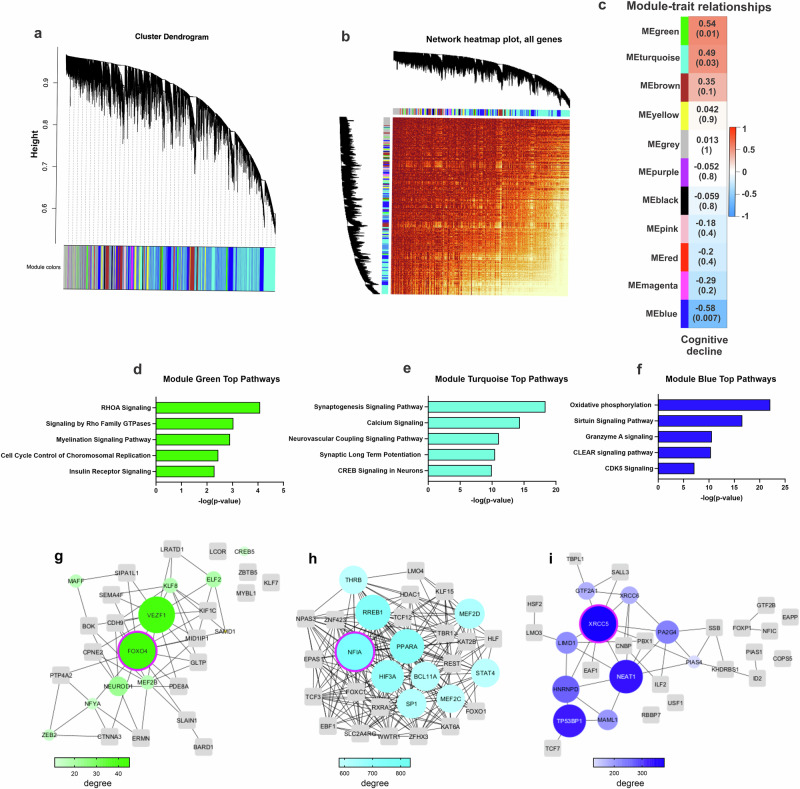
Identification and analysis of co-expression modules related to cognitive decline in AD. **a** WGCNA dendrogram of average hierarchical clustering of genes from twenty AD ITG brain tissue samples. The branches represent individual genes and color bands represent the assigned modules. Genes in the same color band (or module) have similar expression patterns across samples. **b** In the Topological Overlap Matrix (TOM) heatmap of the AD samples, strength of topological overlap is represented by the color shade; the lighter color represents lower overlap, and the progressively darker red color represents higher overlap between the genes. **c** Module-trait relationship of cognitive decline correlations in AD cases. Three modules: green, turquoise and blue significantly correlated with the rate of cognitive decline across samples. The color scale (blue-red) denotes correlation values from −1 to 1 with *P* values shown in parenthesis. **d**–**f** Bar charts showing significantly enriched IPA canonical pathways in the green (left), turquoise (middle) and blue (right) modules. **g**–**i** Network visualization of top key drivers in trait-associated modules. The top 30 genes in the green (left), turquoise (middle), and blue (left) modules are displayed. The size and color intensity of the top 10 genes (circular nodes) are arranged in decreasing order of degree scores. Genes with highlighted borders (magenta) represent the key drivers identified as central regulators within each module network.

We performed weighted gene co-expression network analysis (WGCNA) [41] using gene expression data from AD ITG samples to identify clusters (modules) of co-expressed genes (Fig. 8a, b) and investigate how groups of co-expressed genes are associated with disease progression. The eigengenes of three modules (green, turquoise, and blue) showed significant correlations with the rate of cognitive decline (Fig. 8c). Interestingly, the green and turquoise modules were significantly positively correlated with cognitive decline (green, r = 0.54, P = 0.01; turquoise, r = 0.49, P = 0.03), and were enriched for several signaling pathways involved in regulating cell migration, proliferation, survival and metabolism such as Ras homolog gene family member A (RhoA) signaling, myelination signaling pathway, cell cycle control of chromosomal replication, insulin receptor signaling and pathways regulating synaptic plasticity including calcium signaling, synaptic long-term potentiation and CREB signaling in neurons (Fig. 8d, e). Importantly, the blue module significantly negatively correlated with the rate of cognitive decline (r = −0.58, P = 0.007) and was enriched for several neurobiological pathways important for neuronal development, connectivity, plasticity, and differentiation such as oxidative phosphorylation, sirtuin signaling pathway, granzyme A signaling and CDK5 signaling (Fig. 8f). In sum, WGCNA analysis reveals that signaling pathways in the ITG crucial for synaptic and neuronal plasticity, contribute to the varying degrees of tau seeding-mediated cognitive decline observed in AD patients.

To uncover the causal regulators within the co-expression networks, we performed Key Driver analysis using Algorithm for the Reconstruction of Accurate Cellular Networks-Adaptive Partitioning (ARACNe-AP) [27] on module-based unweighted co-expression networks generated (Fig. 8g–i, Supplementary Table 5). The analysis identified FOXO4, a member of the FOXO family of transcription factors, as the top key driver in the green module. FOXOs are strongly implicated in neurodegenerative diseases, regulating processes associated with cognitive decline, vascular dysfunction, behavior disorders, and neuronal damage [42, 43]. Specifically, FOXO4 transcriptional activity is crucial for maintaining cellular homeostasis and coordinating stress response pathways, including oxidative stress resistance, apoptosis, autophagy, and cell cycle regulation [44]. These processes are particularly relevant in the context of aging and age-related diseases including AD, where dysregulation of these pathways contributes to disease progression. Consequently, FOXO4 may function as a key regulator of cellular resilience and stress adaptation in AD, highlighting its potential role in mitigating disease progression. NEUROD1 was identified as another high-ranking key driver within the green module. As a neural transcription factor, NEUROD1 is crucial for adult neurogenesis, neuronal plasticity and the survival of neuronal progenitors [45, 46]. Notably, NEUROD1 has been used successfully to reprogram reactive glial cells into functional cortical neurons in an AD mouse model [47]. These findings suggest that NEUROD1 could serve as a potential therapeutic target in regenerative medicine, offering the ability to repair and restore lost neuronal function in the diseased brain.

In the turquoise module, NFIA emerged as the top key driver. NFIA is a key transcriptional regulator involved in the early stages of astrocyte differentiation [48] and regulation of astrocyte responses [49]. Astrocytes induced by NFIA exhibit neuroprotective properties, promote synaptogenesis, and display functional calcium transients [50]. The transcriptomic profile of AD-associated reactive astrocytes shows significant downregulation of homeostatic genes enriched for transcription factors, including NFIA, suggesting that the loss of homeostatic functions in reactive astrocytes may contribute to AD pathogenesis by impairing neuronal support and synaptic maintenance [51]. RREB1, another high-scoring key driver in the turquoise module is a zinc finger transcription factor that acts downstream of RAS signaling [52]. It regulates diverse biological processes, including transcriptional regulation, DNA repair, cell proliferation, and microtubule dynamics [53]. By positively modulating genes associated with the microtubule network, RREB1 contributes to maintaining neuronal architecture and promoting dendritic branching, processes that are often disrupted in tauopathies like AD [54].

The top key driver in the blue module was XRCC5 (Ku86), a critical component of the non-homologous end joining pathway, which facilitates the repair of DNA double-strand breaks (DSBs). XRCC5 plays a central role in maintaining genomic stability through its involvement in DSB repair, telomere maintenance, and transcriptional regulation [55–57]. Dysregulation of DSB repair processes has been implicated in AD, where an accumulation of DSBs and alterations in repair proteins may represent an early event contributing to neuronal damage and cognitive decline. Given that aging, a major risk factor for AD, is associated with sustained DSB accumulation, these findings suggest that XRCC5 could be a key mediator of genomic instability in AD. Moreover, tau pathology, through phosphorylation and microtubule destabilization, may exacerbate DSB repair deficits, further compromising neuronal survival. These results highlight XRCC5 as a potential link between genomic instability, tau-driven pathology, and mechanisms underlying neuronal vulnerability in AD. NEAT1, a highly conserved long non-coding RNA (lncRNA), was identified as a high-ranking driver in the blue module. This lnRNA regulates hippocampus-dependent memory formation and memory-associated gene expression [58]. NEAT1 has been implicated in endocytosis-related gene regulation and amyloid beta oligomer uptake by glial cells [59]. Furthermore, it modulates cytoskeletal components through interactions with microRNAs, as demonstrated in a mouse model of AD [60]. These findings suggest that NEAT1 may play a multifaceted role in AD pathogenesis by influencing memory-related processes, amyloid beta dynamics, and cytoskeletal integrity, potentially driving disease progression.

## Discussion

In this study, we describe a detailed investigation into the biochemical and biophysical properties of tau present in the ITG and PFC brain regions of patients with AD who had varying disease progression rates. Our studies in tau biosensor cells revealed that the seeding properties of biologically active, soluble tau in both ITG and PFC brain lysates highly vary between individual patients. For the first time, this study demonstrates that tau seeding specifically in the ITG, a brain region where accelerated tau propagation initiates, may be responsible for the progressive dementia seen in patients as higher seeding is associated with faster cognitive decline. Our data suggest that AD patients with greater longevity, probably not independent of age of disease onset, had lower levels of bioactive, phosphorylated prion-like tau in the ITG at the time of death compared to patients who died at younger ages. These findings further highlight a potential role for the ITG in facilitating widespread, connectivity-based, prion-like propagation of seed-competent tau into neocortical regions including the PFC that may dictate the clinical course of the disease.

The structural and conformational changes induced by biochemical transformations of the tau protein such as post-translational modifications (PTMs) can occur early in the course of AD before NFTs can be identified [61, 62]. We envision that the brain region-specific distribution of these alterations can be closely correlated with neuronal loss and cognitive decline. Furthermore, individual variations in the patterns of tau PTMs, such as phosphorylation at specific sites, may influence the biophysical properties of the protein, including oligomerization and seeding propensity. We postulate that within different brain regions of AD patients with varying cognitive decline, misfolded, soluble tau adopts multiple biologically active conformations. These conformers, linked to variations in the hyperphosphorylated tau profile and tau seeding activity among patients, can assemble into insoluble aggregates with prion-like properties. It has been suggested that inclusion morphology is an indicator of distinct tau prion strains [21, 63], and to our knowledge, this is the first study to show that disease associated ensemble or cloud of conformations exist within patients displaying heterogeneity in tau seeding activity. Additionally, specific types of morphology tend to predominate based on the seeding potential of the samples. These findings suggest that tau conformers may not be uniform, but rather exhibit strain- and patient-specific variations in seeding and propagation. This raises the possibility that these differences contribute to the heterogeneity of cognitive decline observed in AD patients.

Besides differences in morphology, the various types of tau aggregates may be differentiated by the isoform and phosphorylation state of tau. We investigated whether variations in tau aggregate morphology could be linked to the heterogeneity of p-tau profile among AD patients using a panel of antibodies targeting specific regions of the protein. It has been proposed that tau undergoes sequential phosphorylation which is linked to NFT development and the progression of AD [64]. Particularly worth noting is that the epitope defined by phosphorylation of S396 in the C-terminal region of tau has been heavily implicated in AD-associated tau pathology [65]. Although phosphorylation status at a single site does not represent all phosphorylation events, S396 at the C terminus tail, a region with the highest frequency of phosphorylation [66], provides a reliable initial marker of the pathophysiological phosphorylation profile of tau. Previous studies suggest that abnormal tau phosphorylation at S396 is associated with reduced binding to microtubules [65], C-terminal tau cleavage [67] and conformational changes [68] that facilitate its aggregation and propagation. Notably, S396-tau phosphorylation is prevalent in intraneuronal lesions across different brain regions and Braak stages, spanning early to late AD [69]. Furthermore, pS396 tau is enriched in mature and extracellular ghost tangles, recognized as sources of seeding species in advanced stages of AD [70] suggesting its potential contribution to the spatial-temporal propagation of tau pathology. S396 tau phosphorylation has also been implicated in long-term depression (LTD) of synaptic transmission [71], highlighting its functional significance in synaptic plasticity necessary for cognitive function. We found a strong positive correlation between this phosphoresidue and tau seeding in both ITG and PFC brain regions. Additionally, a positive correlation between S396 p-tau levels in the ITG and PFC suggests that elevated p-tau in the ITG may act as “seeds,” promoting the formation and accelerated spread of pathological, hyperphosphorylated tau to interconnected regions such as the PFC, potentially exacerbating disease progression. While S396 offers a focused initial assessment, we extended our evaluation to include additional phosphorylation sites, allowing a more comprehensive understanding of overall tau phosphorylation in the context of disease progression. Other disease-related tau sites hyperphosphorylated in AD include T181 and T217, well-established targets in the CSF and plasma biomarkers field [72, 73]. The phosphorylation at another critical site, T231 occurs prior to overt filament formation [64, 74] and is amongst the consistently increased tau PTMs that determine disease severity level. [62] Moreover, modification of phosphorylation at sites S396/S404, one of the earliest events in AD, can induce synaptic failure [75], intracellular aggregation [76] and cognitive deficits in AD [65]. This study was designed to quantitatively analyze spatial patterns of tau phosphorylation at multiple residues (T181, T217, T231, S396 and S396/S404) important for tau propagation, particularly within the ITG and PFC regions during AD progression. We predict that modifications on tau structure and function may influence its seeding potential with elevated phosphorylation at these crucial tau sites (proline-rich mid-domain, microtubule binding region and the C-terminal domain) associated with increased seeding and spread and subsequent faster cognitive decline. Among many stable forms of tau, hyperphosphorylated HMW-tau is considered the most efficient at transducing recipient cells and seeding [19, 35]. Our data revealed different phosphorylation profiles of the analyzed p-tau sites in the two brain regions. These findings suggest, for the first time, that although phosphorylation of HMW-tau at specific residues, namely T217, T231, S396 and S396/404, but not T181, in both brain regions significantly correlate with seeding, these p-tau sites, specifically in the ITG, potentially contribute to cognitive decline in AD patients. In line with these observations, several studies have suggested that p-sites including T217 and T231 could be more strongly related to AD progression and disease-related tau pathology than T181 in both the CSF and brain [77–79]. Although previous studies have also shown reactivity at 130 kDa in both control and AD human brain samples with the same pT181 antibody [80, 81], it is unclear whether the 130 kDa pT181 band reflects non-specific binding or true pT181-tau species. It could also indicate the presence of pT181 and other proteins of similar molecular weight. Future analyses incorporating specificity assessments of the pT181 antibody would be beneficial for examining potential correlations with tau pathology measures.

Interestingly, while various forms of tau, including insoluble fibrils and soluble hyperphosphorylated HMW-tau, have been linked to tau transmission, recent findings suggest that even smaller, soluble forms such as monomers can also be a seed-competent form [82, 83]. Furthermore, during tauopathy pathogenesis, abnormal and enhanced proteolysis of hyperphosphorylated tau alters tau turnover and generates neurotoxic fragments that can have an increased propensity to be phosphorylated and accumulate due to inefficient clearance mechanisms, thereby exacerbating tau aggregation [84–86]. Tau fragments can also interact with full-length tau to generate soluble, HMW tau species that interfere with axonal transport, missorting of synaptic proteins and induce severe tauopathy-related phenotypes in vivo [87]. Recent research suggests that proteolytic fragments of tau can spread between cells and form aggregates, making them potential candidates for new biomarkers to track disease progression [88–90]. These studies lend further support to our findings that phosphorylation of LMW-tau (monomers and proteolysis products) in high seeders is elevated relative to low seeders at all epitopes tested implicating that these hyperphosphorylated LMW-tau species are also potentially involved in cellular mechanisms of cognitive decline.

Although our findings suggest that tau phosphorylation at specific sites correlates with seeding activity, additional PTMs may be required to fully facilitate tau aggregation, and alternative, non-causal models of this relationship are plausible. The types, site-specific localization, and degree of PTMs may regulate tau seeding and propagation propensity. Tau may initially undergo phosphorylation, which predisposes it to aggregate, and subsequent C-terminal cleavage, elevated phosphorylation in the proline-rich region, and modifications in the microtubule binding domain, such as acetylation and ubiquitination, may further enhance its propensity for fibrillization [66]. Additionally, glycosylation by steric hindrance [91, 92] and prolyl isomerization by facilitating protein phosphatase 2A (PP2A) activity [93] reduce tau phosphorylation and aggregate formation. Other tau PTMs, including glycation, nitration, and polyamination, may render hyperphosphorylated tau more resistant to degradation and increase its sequestration within NFTs, where it can undergo further abnormal ubiquitination [94, 95]. The observed correlation between phosphorylation and seeding may, therefore, reflect underlying structural alterations or combined effects of multiple PTMs, rather than a direct causal relationship.

Differences in microtubule binding properties of tau isoforms have been well-established but several studies have determined additional isoform-specific differences such as templated seeding potential, intra-neuronal re-localization during progression of NFT pathology, and phosphorylation capability [96–98]. In AD, there is evidence of impaired 3R/4R isoform tau ratio in the tangle bearing neurons and aggregation and deposition of 3R-tau may be associated with more advanced stages of the disease [99]. The fact that altering the isoform ratio is associated with the disease state highlights the importance of maintaining the 3R/4R tau balance in healthy neurons. Our findings reveal, for the first time, that AD ITG brain samples contain varying levels of specific HMW-3R and 4R tau conformers, potentially involved in the spread of tau pathology and cognitive decline in patients. We observed a higher fold increase in HMW-3R than 4R-tau in high seeders compared to low seeders resulting in a significant increase of 3R-tau/4R-tau ratio. It is possible that the isoform imbalance may promote microtubule instability and drive the formation of phospho-tau aggregates in neurons, leading to neurofibrillary degeneration. Additionally, LMW-3R and 4R-tau also increased in high seeders emphasizing their potential contribution to disease progression. We propose that distinct and stable LMW-tau isoforms offer a suitable substrate for phosphorylation and formation of larger, toxic assemblies that may serve as highly specific templates to trigger intracellular aggregate formation. Our study suggests that although larger tau assemblies such as soluble, phosphorylated HMW-tau are considered primarily involved in tau propagation linked to cognitive decline, the potential involvement of LMW tau species, especially in early stages, cannot be excluded. If such bioactive LMW-tau can be detected and measured early enough in patients, it may allow treatments for AD and related tauopathies before the development of overt neuropathology.

Growing evidence suggests that soluble forms of tau, especially oligomers, are toxic to synapses and the progression of tau pathology through synaptically connected neurons closely correlates with synapse loss and cognitive symptoms [100]. Our results showed disease progression and tau pathology-related changes in the levels of the synaptic markers, PSD-95 and synaptophysin, with decreased levels associated with increased seeding and faster cognitive decline. Additionally, changes in synaptic function and number due to, for example, neuronal loss, may be reflected in presynaptic and postsynaptic protein expression measured in the extracted tissue. This study provides the first evidence, to our knowledge, of the significance of soluble HMW tau isoforms and specific p-tau (T231, S396 and S396/S404) in relation to synaptic loss observed in the ITG and PFC brain regions associated with tau propagation and cognition. These findings further highlight the crucial role of specific regions within the tau protein, notably the microtubule binding region and C-terminal domain, in its spread within the brain and its potential to induce synaptoxicity. By delineating the association between particular HMW-tau forms and synaptic loss, this study contributes to a deeper comprehension of mechanisms underlying cognitive decline in AD.

The present study also provides RNA-seq transcriptome analyses of the ITG tissue from AD subsets (high and low seeders) and cognitively unimpaired (control) subjects. We aimed to identify the most prominent alterations in transcriptional networks, molecular pathways and biological functions that are associated with tau seeding in the ITG, potentially responsible for its accelerated propagation into connected regions during disease progression. Current evidence suggests that neuroinflammation has a prominent role in AD pathogenesis and progression [101] and patients with AD having relatively high levels of pathological tau showed more severely impaired synaptic plasticity and faster cognitive decline [102]. Our global analysis revealed the enrichment of gene signatures indicative of neuroinflammation while genes associated with synapses were underrepresented in high seeders compared to control and low seeders. We predict that the secretion of pro-inflammatory mediators by elevated immune cells may cause synaptic dysfunction, neuronal death, and inhibition of neurogenesis in agreement with previous data [103]. Additionally, these cells may contribute to dysregulated microcirculation and blood-brain barrier damage that may in turn cause synaptic and neuronal dysfunction and cognitive impairment [104]. Furthermore, our analysis identified top candidate genes related to cognitive decline in the ITG brain region primarily involved in multiple signaling pathways crucial for various cognitive functions, including metabolic homeostasis, myelin production, calcium signaling, immune pathways, neurogenesis, vasculogenesis, and cell survival. Therefore, in our study cohort, we showed for the first time that heterogeneity in cognitive decline associated with tau propagation could, at least in part, be related to tau seeding-mediated failure of neural mechanisms in the ITG, practically a loss of neural and synaptic plasticity. Furthermore, the transcription factors and regulatory molecules identified in our study, including FOXO4, NFIA, and XRCC5 (Ku86), represent critical regulators of pathways implicated in tau pathology in AD. Dysregulation of FOXO4 and NFIA, essential for maintaining cellular resilience and homeostasis, may exacerbate oxidative stress, impair autophagic pathways, and reduce neuronal support, promoting tau accumulation and spread. Similarly, dysregulation of XRCC5 (Ku86) connects genomic instability to neuronal vulnerability, as deficits in DNA repair may increase tau-driven damage. Collectively, these findings emphasize the complex relationship between transcriptional regulation, genomic stability, and cellular resilience, presenting promising therapeutic targets. Restoring their functions could mitigate tau pathology, preserve synaptic integrity, and slow cognitive decline in AD.

The differential correlations between tau seeding in the ITG and the PFC with cognitive decline suggest region-specific roles of tau pathology in disease progression. Given its early involvement in disease, the ITG is likely a critical site for tau accumulation and spread that drives cognitive impairment. Higher tau seeding activity in the ITG thus correlates with more rapid cognitive decline, reflecting its central role in the neurodegenerative process. The PFC is typically involved later in the disease course, with tau pathology accumulating more gradually. While evidence supports the hypothesis that seeding ability correlates with the spread of tau pathology, an alternative explanation should also be considered: the spread may, in fact, represent a slower rate of accumulation of seed-competent tau in the PFC. Tau pathology in the PFC may reflect slower, cumulative damage rather than active tau propagation, with several factors potentially contributing to this interpretation. The underlying mechanisms that govern the pattern of pathological tau propagation remain unclear, but may include neuronal vulnerability factors, high metabolic rates that increase susceptibility to oxidative stress, cellular and regional connectivity, and independent processes such as spread of neuroinflammation [105]. Genes linked to exocytosis, endocytosis, synaptic vesicle function, protein clearance, and intracellular transport may also play direct roles in tau propagation [106]. The spread of tau pathology aligns with selective neuronal vulnerability to pathological processes, possibly progressing through a prion-like mechanism. Connected cells may exhibit varying sensitivity to prion-like mechanisms of seed release, uptake, and aggregation. Independently of prion-like mechanisms, some cells may effectively degrade aggregates for extended periods before becoming affected, a factor that may explain the age-related dependency observed in AD. When the balance between degradation and aggregation shifts toward aggregation, ordered assemblies of tau may then be able to spread. Furthermore, regions with advanced tau pathology, as characterized by Braak staging in post-mortem analyses, typically exhibit higher tau seeding, suggesting a possible connection between pathological spread of tau and its propensity to seed further aggregation [107, 108]. Variation in seeding activity between patients at early and advanced disease stages may reflect differences in tau aggregate burden, cell death, or ghost-tangle formation at later stages. The early and widespread detection of tau seeding supports the prion-like propagation model, suggesting that seeding may drive progressive tau accumulation and associated neurological dysfunction in AD.

In sum, this study highlights the potential molecular drivers of widespread neocortical tau propagation that begins in the ITG brain region and possibly drive disease progression. Intriguingly, we found differences in biochemical and biophysical features of both HMW and LMW-tau and transcriptomic signatures associated with tau seeding in the ITG and clinical disease progression that varies substantially between patients. Hereby, the current study sheds light on the complexities of AD progression that will allow further progress in patient stratification to improve clinical trials and develop personalized treatments.

## Supplementary information

Supplementary Figures

Supplementary Tables

## Data Availability

Data supporting the findings of this study are available in the main text and supplementary information files. All other data generated in the study are available from the corresponding author on request. The RNA-seq datasets generated for the current study have been deposited in GEO under the accession ID: GSE282910.

## Code availability

All codes used in this study will be made available upon request from the corresponding author.
