# Supplementary material for: Neocortical tau propagation is a mediator of clinical heterogeneity in Alzheimer’s disease
Source: Mol Psychiatry. 2025 Apr 16;30(9):4194–213. doi: 10.1038/s41380-025-02998-y (PMC12501883; doi:10.1038/s41380-025-02998-y)

Supplementary Information

Supplementary Fig. 1 Age of disease onset and age at death correlate with ITG but not with PFC tau seeding activity. Tau seeding activity of ITG and PFC brain lysates were correlated with the age of disease onset (**a**) and age at death (**b**). Correlations were performed using a two-tailed Spearman's rank non-parametric correlation, *r* and *P* values are indicated on the plots. n=20 individual AD subjects

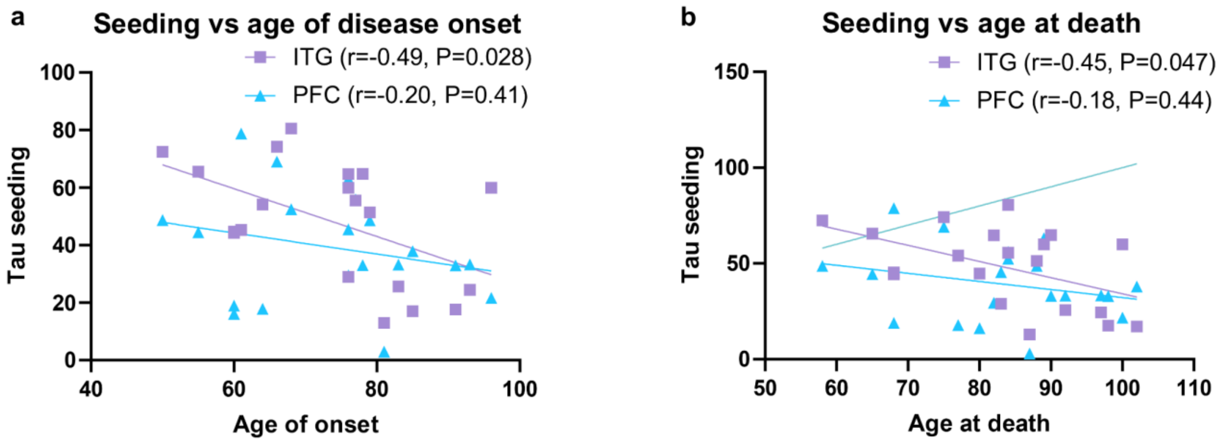

Supplementary Fig. 2 Correlation of HMW-phosphorylated tau in ITG versus PFC. Intensity of phosphorylation of phospho-sites T181, T217, T231, S396 and S396/S404 in the ITG and PFC were correlated. Two-tailed Spearman’s rank non-parametric correlation tests were used, and *r* and *P* values are indicated on the plots. n=20 individual subjects

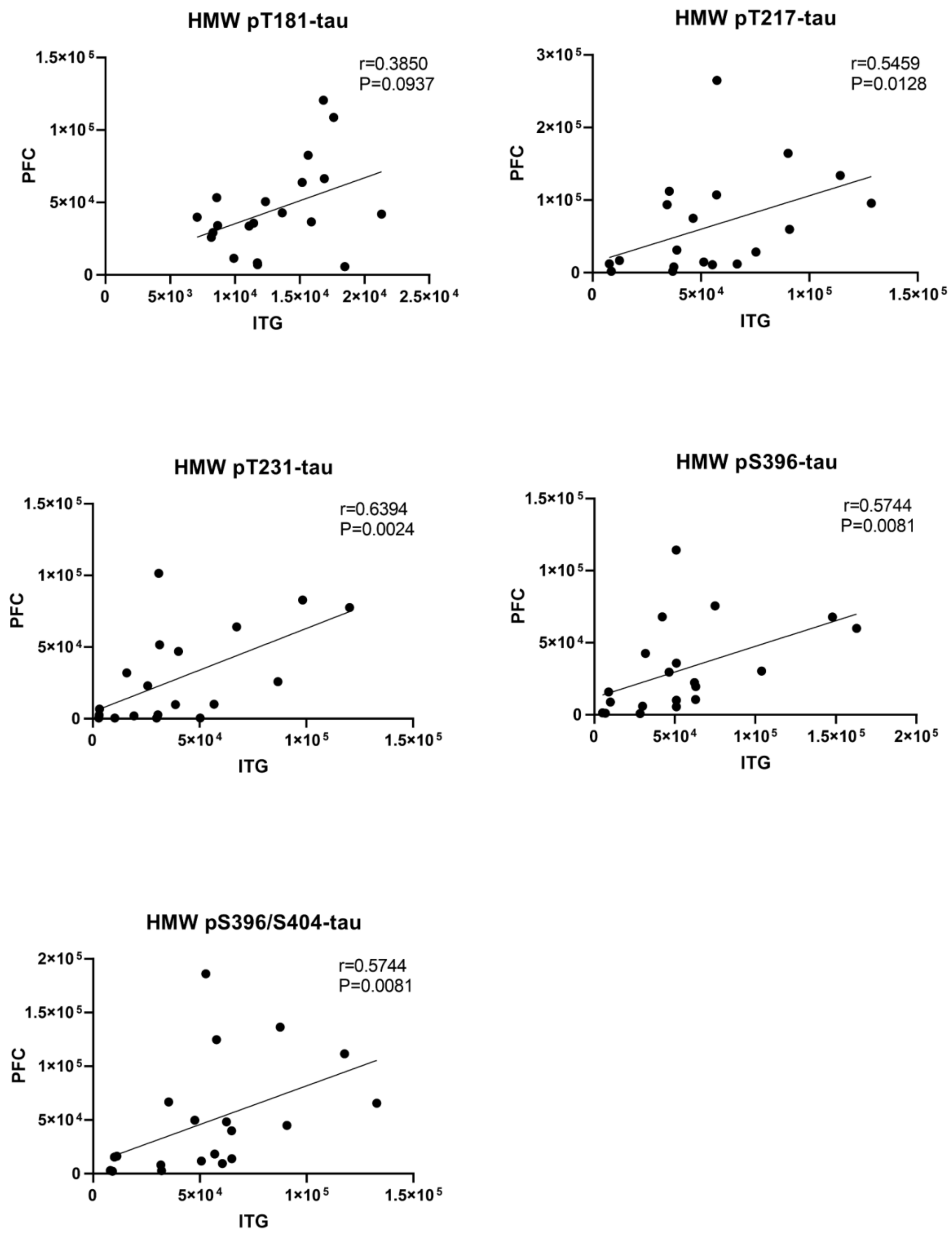

Supplementary Fig. 3 Correlation of HMW-phosphorylated tau and tau isoforms in PFC AD brain versus rate of clinical disease progression. Intensities of HMW-tau phosphorylation at phospho-sites T181, T217, T231, S396 and S396/S404, HMW total tau (Tau13), 3R and 4R tau isoforms determined by western blots were correlated with rate of cognitive decline (**a**, **b**) and synaptic markers PSD-95 and synaptophysin (**c**, **d**). Two-tailed Spearman's rank non-parametric correlation tests were used, and *r* and *P* values are indicated on the tables. n=20 individual subjects

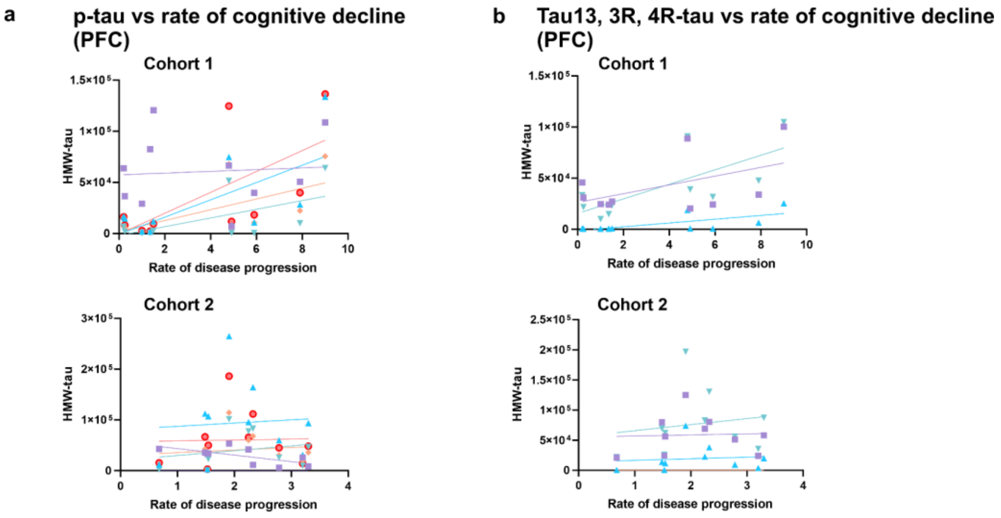

Phosphoresidue(s)	Cohort 1 r	Cohort 1 P	Cohort 2 r	Cohort 2 P
T181	0.1636	0.6567	-0.6970	0.0306
T217	0.3697	0.2957	0.05455	0.8916
T231	0.3189	0.3656	0.3455	0.3304
S396	0.5273	0.1231	0.2485	0.4918
S396/S404	0.6848	0.0347	-0.01818	0.9730

Total tau & tau isoforms	Cohort 1 r	Cohort 1 P	Cohort 2 r	Cohort 2 P
Tau 13	0.1273	0.733	0.103	0.785
3R	0.5758	0.0883	0.3212	0.3679
4R	0.6727	0.039	0.3697	0.2957

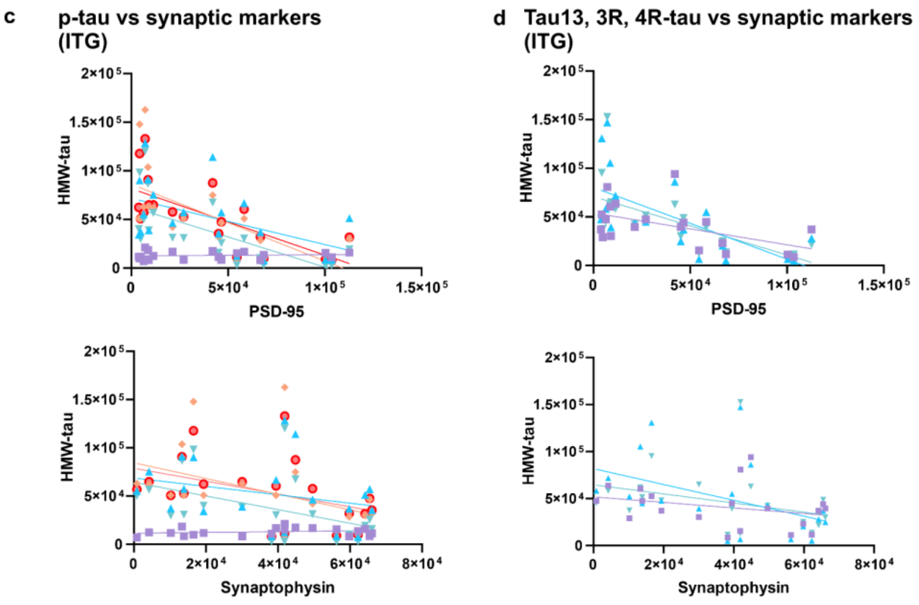

Phosphoresidue(s)	PSD-95 r	PSD-95 P	Synaptophysin r	Synaptophysin P
T181	0.1158	0.6269	0.1865	0.4312
T217	-0.4391	0.0528	-0.3218	0.1665
T231	-0.7895	<0.0001	-0.5308	0.0160
S396	-0.7654	<0.0001	-0.5188	0.0191
S396/S404	-0.7609	<0.0001	-0.4571	0.0427

Total tau & tau isoforms	PSD-95 r	PSD-95 P	Synaptophysin r	Synaptophysin P
Tau 13	-0.5474	0.0125	-0.3383	0.1445
3R	-0.7534	0.0001	-0.5579	0.0106
4R	-0.7188	0.0004	-0.4000	0.0806

Supplementary Fig. 4 Transcriptional dysregulation of synaptic organization in AD ITG. (**a**, **b**) Sunburst plots of enriched cellular component terms in high versus control and high versus low seeders; colors are based on the -log 10 Q-values (FDR corrected raw *P*-values) for enriched synaptic GO terms. The bar plots highlight top six enriched cellular component terms. (**c**, **d**) Manhattan plots of GO cellular processes enriched in high seeders versus control and high versus low seeders for each cluster obtained using g:profiler. Colored circles denote significant terms (thresholds: g:SCS significance < 0.01; GO term size 5-350). Top 5 nonredundant terms are highlighted. (**e**) Functional enrichment map. Nodes represent gene sets of GO cellular processes; each node is color coded by cluster to illustrate shared and unique contributions

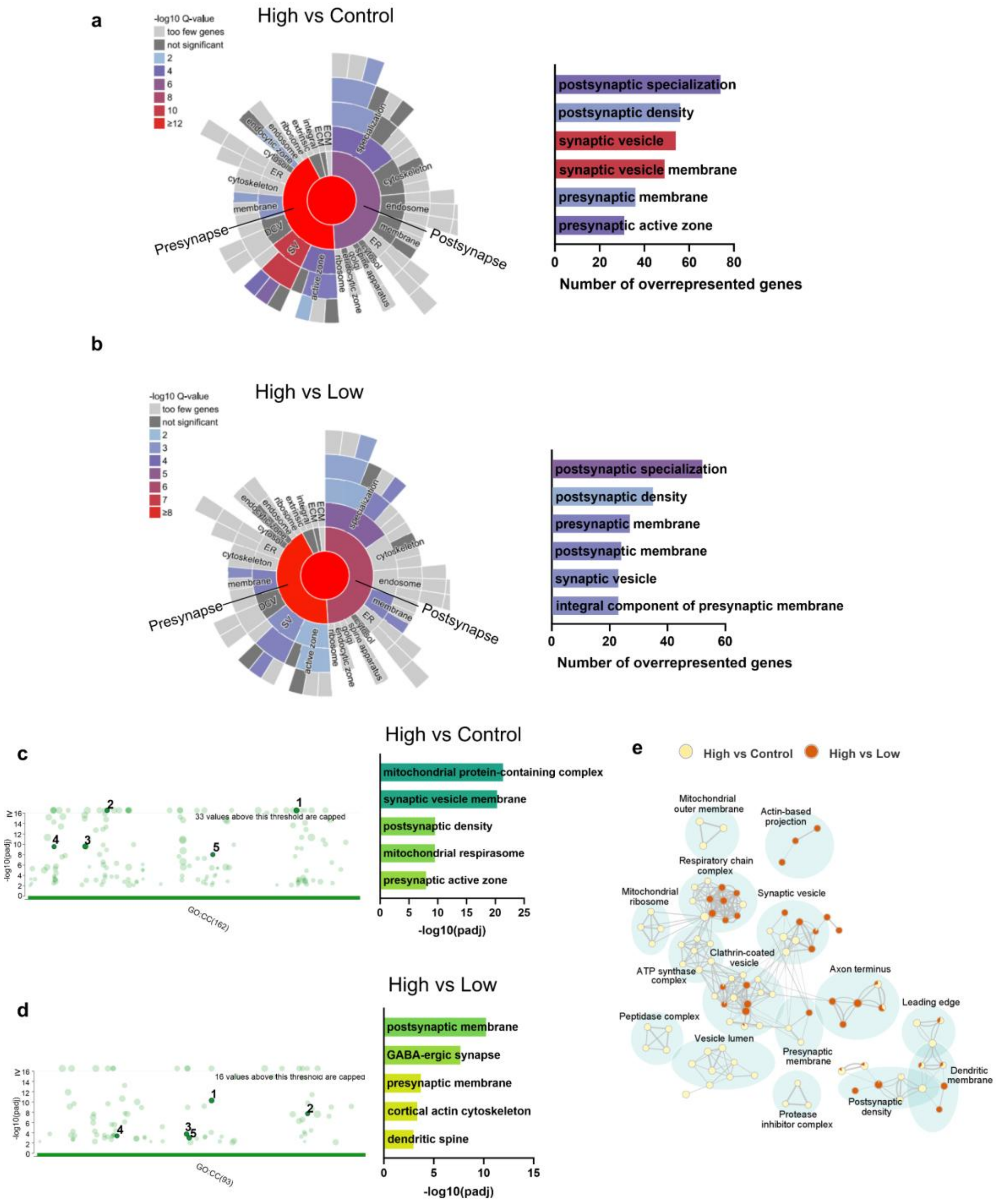

Supplement: Supplementary file 1 — Supplementary Figures [file 41380_2025_2998_MOESM1_ESM.pdf]
